# Identification two novel nacrein-like proteins involved in the shell formation of the Pacific oyster *Crassostrea gigas*

**DOI:** 10.1007/s11033-014-3298-z

**Published:** 2014-03-02

**Authors:** Xiaorui Song, Xiaotong Wang, Li Li, Guofan Zhang

**Affiliations:** 1 National & Local Joint Engineering laboratory of Ecological Mariculture, Institute of Oceanology, Chinese Academy of Sciences, Qingdao, 266071 Shandong China; 2 Graduate University of Chinese Academy of Sciences, Beijing, 100049 China

**Keywords:** Nacrein-like proteins, Shell biomineralization, *Crassostrea gigas*, Calcite fractions

## Abstract

Nacrein-like proteins have carbonic anhydrase (CA)-like domains, but their coding regions are flanked by inserted repeat sequence, such as Gly-X-Asn. Reportedly, nacrein-like proteins show the highest similarity to human carbonic anhydrase 1(α-CA1), possess CA catalytic functions, and play a key role in shell biomineralization. In the present study, two novel nacrein-like proteins were firstly identified from the shell-forming mantle of the Pacific oyster *Crassostrea gigas*. With numerous analyses, it was identified and characterized that both the nacrein-like proteins F1 and F2 were secreted and most closely related to the nacrein-like protein of California mussel *Mytilus californianus* via phylogenetic analysis. RT-PCR analysis showed that the nacrein-like proteins F1 and F2 were expressed in multiple tissues and the expression levels remarkably rose after entering the spat stage, which were basically consistent with the increase of calcite fractions in the total shell volume. Surprisingly, the Gly-X-Asn repeat domain, which is distinctive in most nacrein-like proteins, was absent in the two newly identified nacrein-like proteins in *C. gigas* and replaced with a series of acidic amino acids (D/E). Regardless, nacrein-like proteins in mollusks seem to be vital to the deposition of calcium carbonate and likely perform diverse functions.

**Electronic supplementary material:**

The online version of this article (doi:10.1007/s11033-014-3298-z) contains supplementary material, which is available to authorized users.

## Introduction

The mollusc shell is a remarkable model of matrix-mediated mineralization performed outside the living tissues. The shell organic matrix mainly consists of proteins, chitin, and polysaccharides that can precisely self-assemble, which is considered as a key role in crystal polymorphism, nucleation, growth, and termination to form the final texture of the shell [1]. Thus, the incredible regulation during shell formation is regarded as a good example to study calcium carbonate biomineralization [2].

Nacrein, the first identified organic matrix component in the Japanese pearl oyster *Pinctada fucata*, was found to be specifically involved in nacreous layer formation in the shell and pearl [3]. Nacrein has a carbonic anhydrase (CA)-like domain that is separated by a Gly-X-Asn (X = Asp, Asn, or Glu) repeat sequence and was found to function as a CA, although its in vitro activity was lower than that of a true CA. Surprisingly, the Gly-X-Asn repeat sequence seemed to act as a negative regulator of calcification in the nacreous layer of *P. fucata* [4]. Nacrein homologs were also identified in the nacreous layer of silver-lip pearl oyster (*Pinctada maxima*) [5], turban shell (*Turbo marmoratus*) [6], the edible Iwagaki oyster (*Crassostrea nippona*), Yesso scallop (*Patinopecten yessoensis*) [7], giant clam (*Tridacna gigas*) [8, 9], and pearl oyster (*P. maxima*) [5, 7, 10]. Although the CA domains of the homologs share high similarity with nacrein, the repeat sequences have slight differences in length and composition. However, current information regarding nacrein-like proteins is largely restricted to the pearl oyster, as investigations on other mollusks have been scanty.

The Pacific oyster *Crassostrea gigas* (Thunberg, 1793) is a marine bivalve belonging to the phylum Mollusca, and has long been as an interesting model for developmental biology. Regarding shell biomineralization, *C. gigas* also can be considered as an experimental model for several unique characteristics. For one thing, except for five small, distinct, well-defined areas consisting of aragonite, the adult shell of *C. gigas* mostly consists of calcite crystals [11, 12], which is significantly different from the *Pinctada* nacre model. For another thing, the inner shell microstructure of *C. gigas* is composed solely of foliated and chalky structures, while it is more diverse in the pearl oyster [13]. Furthermore, based on the extensive analysis of genes implicated in shell formation and mass spectrometric analysis of shell proteins, there is strong evidence that cell is involved in mollusc shell formation [14, 15].

The present study is the first to report the complete sequences of two novel nacrein-like proteins isolated from the shell-forming mantle of *C. gigas* and to describe their structures and evolutionary relationships with all known nacrein-like proteins and CAs already identified in the mollusks. Our results revealed that the diversity in shell organic matrix compositions maybe decide the variety of shell structure.

## Materials and methods

### Biological materials

Wild adult Pacific oysters were collected from the aquatic farm in Laoshan (Qingdao, China) and healthy individuals were selected and cultivated in aerated seawater at 23 °C for several days prior to use.

Larva oysters of different developmental stages were cultured from a hatchery located in Laoshan (Qingdao, China) using an insemination technique modified from the methods described by Fujimura et al. [13]. Eggs from 80 females were dissected, rinsed with filtered seawater, screened through a 90-μm nylon mesh, then resuspended in a 25-l bucket containing seawater at an approximate density of 100,000 eggs/ml. Spermatozoa from five males were rinsed into a 1-l bucket and then added to the egg suspension until the proportion between spermatozoa and eggs reached ~10–15:1. Thus, about 250 million zygotes were obtained and transferred to a 25-m^3^ bucket (density, 30 zygotes/ml) of filtered seawater and incubated at 23 °C. The larvae were observed via light microscopy, harvested at appropriate stages, and then rapidly frozen in liquid nitrogen along with one sample of unfertilized eggs. The salinity of the filtered seawater used in the experiments was ~30 ppt.

### cDNA cloning

Total mRNAs were extracted from the shell-forming mantle of wild adult oyster using Trizol^®^ reagent (Invitrogen) with the manufacturer’s protocol, and quantified using a NanoDrop spectrophotometer (ND-2000/2000C; Thermo-Fisher Scientific), and then stored at −80 °C for further use. The integrity of the RNA samples was also evaluated by agarose gel electrophoresis. Reverse transcription was performed with 1 μg of total mRNAs using the Prime Script RT reagent Kit with gDNA Eraser (TaKaRa) to avoid genomic DNA contamination, and dT-AP served as the reverse transcription primer.

Two sequences described as nacrein-like proteins after sequence homology analysis were retrieved from the oyster genome database. Firstly, two pairs of test-primers were designed based on the two sequences and used to obtain the nacrein-like protein F1 and F2 transcripts. The extracted PCR products were subcloned into a pEASY-T1 vector (TransGen), and positive clones were sequenced. Secondly, the complete 3′ and 5′ end sequences were obtained via nested-PCR using 3′ and 5′ RACE primers, which were designed according to the above nucleotide fragments. In the 3′ RACE nested-PCR, AP/3RACE-F1 and AP/3RACE-F2 were used to obtain a complete 3′ end sequence, including the polyadenylation (poly (A)) signal. The cDNA templates used in the 5′ RACE nested-PCR were required to be purified and add poly (C) to the 5′ end in advance, then primers dg-AP/5RACE-R1 and dg-AP/5RACE-R2 were employed to obtain a complete 5′ end sequence. The PCR products were subcloned and sequenced by the method described above. Finally, the full length cDNA sequences were confirmed using gene-specific primers, which were designed based on the untranslated regions of the 5′ and 3′ terminals, respectively. The synthesis of all PCR primers and cDNA sequencing were completed by Sangon Biotech (Shanghai) and all the primer sequences are listed in Table S1.

### Primary sequence analysis, alignment, and phylogeny

The two full-length cDNA sequences in *C. gigas* were identified as being nacrein sequences using tblastx analysis in NCBI database (http://www.blast.ncbi.nlm.nih.gov/) and then analysed in silico using various tools from the Centre for Biological Sequence Analysis database (http://www.cbs.dtu.dk/services/). The deduced amino acid sequences were compared together with six nacrein-like proteins reported in mollusks and CA1 of *Homo sapiens* using the ClustalX (www.clustal.org/).

Sequences of 18 nacrein-like proteins from bivalves, one nacrein-like protein from gastropod, and 16 CAs from mollusks were aligned using the ClustalX. The alignment was manually modified using Bioedit software and Gly-X-Asn repeats in all nacrein sequences were removed for the alignment. The Phylogenetic Estimation using Maximum Likelihood algorithm with a BIONJ-defined starting tree [16], was used to obtain maximum likelihood trees under the WAG amino acid substitution matrix model and the parameter values previously estimated by ProtTest version 2.4, which was generally used to determine the best-fitting model for protein evolution [17].

### Transcript quantification by real-time PCR

Real-time PCR analyses were both performed on cDNAs from seven tissues of three adults *C. gigas* (right mantle, left mantle, gill, gonad, haemocyte, adductor muscle and gut) and seven stages of *C. gigas* (egg, trochophore, D-shaped larvae, umbo larvae, pediveliger, spat and juvenile). Total mRNAs (1 μg) were extracted and quantified as described above. For each gene, RT-primers were designed (Table S1) and tested using serially diluted total mRNAs (1, 1:10; and 1:100) according to the original concentration. The gel pictures were analysed to verify the specificity of the amplified products. Real-time PCR was performed using an ABI 7500 Fast instrument and 7500 software version 2.0.1 (Applied Biosystems, USA). Each sample from the three specimens was amplified in triplicate and a negative control was analysed in parallel for each gene. Elongation factor 1 alpha (EF1-α; Accession number: AB12206 6) and ribosomal protein S18 (RS18; oyster gene ID: CGI_10008101) were used as reference genes in the adult and larva samples, respectively [18]. The gene expression levels were determined by directly comparing CT values between target genes and reference genes, and the relative quantities were calculated using the ∆∆CT method with the light value as a calibrator. Finally, data were converted to linear form using the 2^−∆∆CT^ method [19].

## Results

### Cloning of two nacrein-like proteins from the Pacific oyster

The nacrein-like protein F1 consists of 1,631 bp encoding 428 amino acids from a 1284-nucleotide open reading frame, ranging from the ATG translation initiation codon to the TAA stop codon. The nacrein-like protein F2 contains a 1287-nucleotide open reading frame encoding 429 amino acids, with the translation initiation codon ATG and the stop codon TGA. The two nucleotide sequences were deposited in the GenBank database under the accession numbers KC563208 and KC563207, respectively.

### Predictive characterization of the two nacrein-like proteins

Blastp analyses clearly showed that the two sequences were members of the α-CA family and each possessed a conserved CA catalytic domain inserted by a repeat sequence of acidic amino acids (D/E) (Fig. S1). In particular, three histidine residues essential for the zinc cofactor binding and 25 active residues possessing a critical function in CA activity both appeared in the two sequences (Fig. S1), indicating that they had an active function in the shell-forming mantle [20]. The primary structure analyses indicated a molecular weight of 49.53 kDa and an iso-electric point of 4.72 for nacrein-like proteins F1, and 49.8 kDa and 6.54 for nacrein-like protein F2 [21]. The signal peptides predicted through the SignalP 4.0 server [22] were both present in nacrein-like proteins F1 and F2 with probable cleavage sites between positions 20 and 21, as well as 22 and 23. Analysis using the TMHMM version 2.0 server for prediction of transmembrane helices showed that F1 and F2 both had a transmembrane region at separate N-terminal sites, which are considered as the secretion signal peptides. The TargetP 1.1 server confirmed that the two proteins are transported through the secretory pathway. An additional simulation using the big-PI predictor server [23] showed that the two proteins have no glycophosphatidylinositol anchor sites. On the whole, the nacrein-like proteins F1 and F2 possess a transmembrane domain and are passed via the secretory pathway without anchor sites, thus the two can be basically considered as secreted CAs. Other particular motifs are also present in the two sequences. The NetPhos 2.0 tool [24] identified 20 putative phosphorylation sites in F1 and 28 in F2. On the basis of the consensus sequences Asn-Xaa-Ser/Thr, the NetNGlyc 1.0 prediction tool revealed four and three potential N-glycosylated sites in F1 and F2, respectively (Fig. S1).

A search of the UniProtKB/Swiss-Prot database revealed that the both two new sequences of *C. gigas* showed the highest similarity with the nacrein-like protein in *Mytilus californianus*, followed by nacrein in *P. fucata*, although the similarity is comparatively low in the Gly-X-Asn repeat domain. What’s more, the two nacrein-like proteins of *C. gigas* share several conserved peptides that are typical among all nacrein-like proteins, such as QSPIN, GSEHS, PMEA, YTYE/A/PGSLT/STPPC, and three histidine residues involved in zinc binding (Fig. S1).

**Fig. 1:**
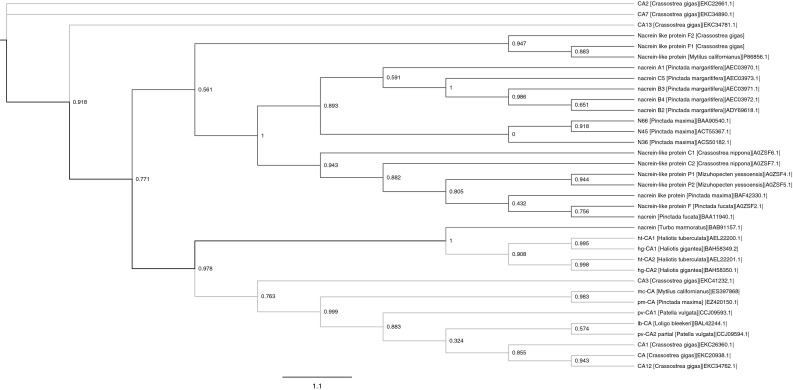
Phylogenetic relationships of 35 nacrein-like protein and CA sequences in mollusks inferred from ML analysis. The test was estimated using a WAG model (α = 0.63) via the PHYML algorithm. The *scale bar* indicates 1.1 substitutions per amino acid site. The target sequences in this study are highlighted in *red*. The blue and purple branches respectively show the nacrein-like proteins in bivalves and nacrein-like proteins in gastropods. The all CAs in mollusks are indicated in *green* branches

A phylogenetic tree was created from the alignment of 35 nacrein-like proteins and CAs among mollusks using the maximum likelihood method (Fig. 1). Overall, the nacrein-like proteins in bivalves were grouped together and separated from nacrein-like proteins in gastropods and all CAs in mollusks. The nacrein-like proteins F1 and F2 identified in the present study were located in the same subcluster, but F1 was grouped most closely with the nacrein-like protein in *Mytilus californianus*. The scale bar indicates 1.1 substitutions per amino acid site.

### Expression of the two nacrein-like proteins in *C. gigas*

**Fig. 2:**
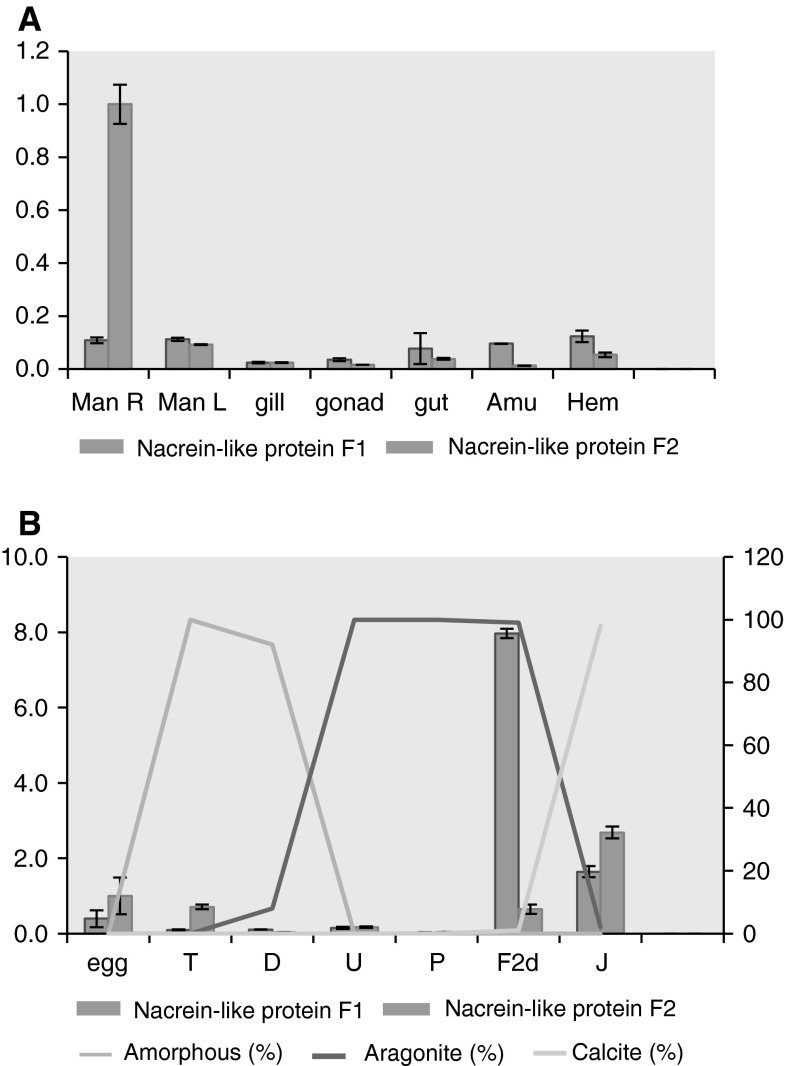
**a** Relative expression levels of nacrein-like proteins F1 (*blue column*) and F2 (*red column*) transcripts in seven tissues (right mantle, left mantle, gill, gonad, gut, adductor muscle, and hematocyte) of the adult *C. gigas* (n ≥ 3). **b** (1) Relative expression levels of nacrein-like proteins F1 (*blue column*) and F2 (*red column*) transcripts in seven stages of the *C. gigas* (n ≥ 3) (refer to the primary axis). The X-axis denotes respectively: the egg, trochophore, D-shaped larvae, umbo larvae, pediveliger, spat, and juvenile stages. (2) The *line* indicates fractions of amorphous tissue and minerals related to the developmental phases of *C. gigas* [33] (refer to the secondary axis). The *olive color* indicates amorphous; *purple* signifies aragonite, and *light blue* denotes calcite

Real-time PCR analyses of different tissues (Fig. 2a) showed that the nacrein-like protein F1 and F2 transcripts were both strongly expressed in the shell-forming mantle of *C. gigas*, although the maximum relative expression levels between the right and left mantles were unequal. The transcript level of nacrein-like protein F2 was also abundant in the hematocyte, but lower expression was detected in the gill and gonad, which was dissimilar to that of a true CA activity, which was the highest in the gill [25]. The expression levels of nacrein-like proteins F1 and F2 transcripts in different stages were similar and peaked in the juvenile and spat stages, respectively (Fig. 2b).

## Discussion

The present report is the first to identify two new shell matrix proteins from the shell-forming mantle of the Pacific oyster *C. gigas*, which were named nacrein-like proteins F1 and F2, respectively. As other nacrein-like proteins in bivalves, the two both exhibit typical catalytic sites of α-CAs with three histidine residues and 25 active cites [20] (Fig. S1). *In silico* analyses of the primary structure revealed the presence of signal peptides and N-terminal transmembrane regions both in F1 and F2, indicating that the two are secreted CAs. It was reported that phosphoryl and saccharide chains might have a close relationship with the biomineralization process, as the phosphorylation of amino acids could change the tertiary structure of the protein and subsequently its interactions with Ca^2+^ [26]. Our results indicated that the two nacrein-like proteins both have several potential phosphorylation sites, which probably involve in shell formation. Most water-soluble matrix proteins contain a number of saccharide chains, as do F1 and F2, indicating that the two should be glycoproteins. Takakura et al. [27] reported that nacrein was a glycoprotein. The sulfites and sialic acid residues on its saccharide chains might provide necessary negative charges to promote the uptake of Ca^2+^. The saccharide chains of F1 and F2 probably have the similar effects.

Nacrein contains a CA domain as well as a Gly-X-Asn repeat domain, and the latter is supposed to interact with calcium carbonate, as indicated by its inhibited precipitation in an in vitro crystallization experiment [4]. However, the two new sequences described herein contain a repeat sequence of acidic amino acids (D/E) instead of the Gly-X-Asn repeat domain. On the one hand, this event conforms to the finding that acidic amino acids and Gly are the major components of an adult oyster shell [28]. On the other hand, this event demonstrates that shell organic matrix compositions vary greatly from species to species on the amino acid level, even within closely related groups [29]. According to the classification proposed by Norizuki and Samata [7], the two new nacrein-like proteins in *C. gigas* can be classified into the type in which protein sequences have low homology with nacrein (*P. fucata*) both in the CA and Gly-X-Asn -repeat domains. The component isolated from *P. fucata* can also be classified into this type [30].

As shown in Fig. 1, nacrein-like proteins from bivalves and gastropods were clearly separated, and the former were further divided into several subclusters, indicating of the obvious distinctions among species. Notably, the nacrein-like proteins in the same species were mostly clustered together, implying that they occurred duplication after speciation. Further, the CAs in bivalves were clustered with nacrein-like proteins and CAs in gastropods, suggesting that the nacrein-like proteins appeared before the divergence of bivalves and gastropods, and the nacrein-like proteins in bivalves seemed to evolve more rapidly than CAs in the earlier phase of bivalve formation, finally becoming an independent cluster, while this event didn’t occur in gastropods. The rapid evolution of nacrein-like proteins in bivalves was presumably caused by the fast changing Cambrian environment, especially the marine chemical composition, which can affect shell biomineralization [31].

RT-PCR analyses showed that the transcripts of nacrein-like protein F1 and F2 were both highly expressed in the shell-forming mantle, but the expression level of nacrein-like protein F1 transcript in right mantle is far higher than in left mantle (Fig. 2a). It is well-known that *C. gigas* lives attached to solid objects through the left shell, thus the differential expression between the left and right mantle may relate to its attachment behavior. We also investigated the expression levels of nacrein-like protein F1 and F2 transcripts during the ontogeny of *C. gigas* (Fig. 2b). In this study, both expression levels could be detected from the egg stage, but exhibited a trend of down-regulation until entering the spat stage, and then reached a maximum value during the juvenile and settlement stages, respectively. Reportedly, biomineralization in oysters begins at an early stage and the oyster shell undergoes polymorphic changes during the entire life cycle of the organism with significant alterations in organic components and protein conformations [32]. In *C. gigas*, amorphous calcium carbonate is initially produced and peaks during the trochophore stage, then begins to transform into aragonite crystals. During the settlement stage, a rapid decrease in aragonite content occurs, followed by an increase in calcite fractions. At the end of the dissoconch stage, a juvenile oyster is finally formed and its shell is composed of 99 % calcite and 1 % aragonite [28, 33]. As shown in Fig. 2b, the up-regulation of the nacrein-like protein F1 and F2 transcripts synchronized with the rise of calcite content. It suggests that the nacrein-like proteins F1 and F2 probably have an intimate relationship with the biomineralization process, especially in the formation of calcite crystals.

Intriguingly, the nacrein-like protein F1 appears not only in the shell-forming mantle but also in the shell matrix, while the nacrein-like protein F2 is only detected in the shell-forming mantle, but not in the shell matrix, although the two are both secreted [15]. It seems that the nacrein-like protein F1 is functional within the shell matrix, as documented in *Pinctada* spp. and in *U. pictorum*, while the nacrein-like protein F2, as exemplified in *H. tuberculata*, is secreted from the mantle, but is not incorporated into the shell matrix [34]. Anyway, to elucidate the molecular mechanisms of nacrein-like proteins participating in the mineralization process, detailed structural and functional analyses are essential.

## Electronic supplementary material

Below is the link to the electronic supplementary material.

Table S1 List of primers used in the present study (DOCX 22 kb)

Fig. S1 Alignments of nine nacrein-like proteins from seven mollusc species and CA1 from *Homo sapiens* were edited using Bioedit software. Conserved amino acids have black (100%) or dark gray (70%) backgrounds, whereas nonconserved amino acids have a white background. The putative active sites of the functional domains in the CA1 (*Homo sapiens*) are indicated by diamonds (◊), among which 25 active sites appear in the nacrein-like proteins of *C.gigas*, including three histidine residues that bind to zinc ions(◊). Signal peptides and the transmembrane region in nacrein-like proteins F1 and F2 are boxed and highlighted in yellow, respectively. Amino acids in pink background indicate putative phosphorylation sites (F1: Ser 14, Thr 2, Tyr 4; F2: Ser 14, Thr 2, Tyr 12); Characters highlighted in green indicate putative N-glycosylation sites (F1: 103, 153, 156, 416; F2: 119, 124, 423). Accession numbers: *Homo sapiens*: CA1/ AAH27890; *Crassostrea gigas*: nacrein-like protein F1, F2 and F3; *Pinctada maxima*: nacrein like protein (pm) /BAF42330.1; Mizuhopecten yessoensis: nacrein-like protein P1/BAF42331.1; *Pinctada fucata*: nacrein/BAA11940.1; *Pinctada margaritifera*: nacrein-like protein C5/AEC03973; *Mytilus californianus*: nacrein-like protein/P86856.1 and *Turbo marmoratus*: nacrein/ BAB91157 (PDF 132 kb)
