# Supplementary material for: Identification two novel nacrein-like proteins involved in the shell formation of the Pacific oyster Crassostrea gigas
Source: Mol Biol Rep. 2014 Mar 2;41(7):4273–8. doi: 10.1007/s11033-014-3298-z (PMC4066178; doi:10.1007/s11033-014-3298-z)
Supplement: Supplementary file 1 — List of primers used in the present study (DOCX 22 kb) [file 11033_2014_3298_MOESM1_ESM.docx]

| Primer names | Primer sequences（5’--3'） | Primer purposes |
| --- | --- | --- |
| **Nacrein-like protein F1** | | |
| Test-F | TGCTGTGGATTTCCCTCG | Test PCR; |
| Test-R | CCCGGTAGTTCCTGCTTT |  |
| 3RACE-F1 | CGGTCCACTTATGAAAGCAGGAAC | 3RACE nested-PCR; |
| 3RACE-F2 | AATGTGGGGACCATCCAGACTTG |  |
| 5RACE-R1 | CTGTCGTAAAACACCAAGTGAGCC | 5RACE nested-PCR; |
| 5RACE-R2 | CTGTAGATTCCCAAACCCCTCCCTG |  |
| Confirm-F | TCCTTTGGGGCTGACTA | Confirm PCR; |
| Confirm-R | AACAAAACACACACTGTCTACG |  |
| **Nacrein-like protein F2** | | |
| Test-F | GGTTGGTGTTTCTAGGTTC | Test PCR; |
| Test-R | CTTCATTGTGGTGGTATTC |  |
| 3RACE-F1 | TGGTCTAGTTGTCATCGGAGTCAT | 3RACE nested-PCR; |
| 3RACE-F2 | CAGCAAGAAGTGCTATAAAGGGAATAG |  |
| 5RACE-R1 | GCTTGCTTTGGTCCTTCTGGCCTCTG | 5RACE nested-PCR; |
| 5RACE-R2 | GTTCCGAACCAACATCCTTCTCCTTGC |  |
| Confirm-F | GCCGAAAATGTAGGGAAAAC | Confirm PCR; |
| Confirm-R | TACCAGCCAGAGCCAAACTAC |  |
| **Real-time PCR primers** | | |
| RT-F1 | GGATGTTCTCCCTTGTGACCAG | RT-PCR primers of F1 transcript; |
| RT-R1 | ACCCCCAGCAATTTCAGTTC |  |
| RT-F2 | TTCCGCTCGATCAAAGGTTC | RT-PCR primers of F2 transcript; |
| RT-R2 | TCTTCTCGGCGTCCTCAACA |  |
| EF-1 α-F | AGTCACCAAGGCTGCACAGAAAG | Internal control primer for adult oysters; |
| EF-1 α-R | TCCGACGTATTTCTTTGCGATGT |  |
| RS18-F | GCCATCAAGGGTATCGGTAGAC | Internal control primer for larva oysters; |
| RS18-R | CTGCCTGTTAAGGAACCAGTCAG |  |
| **Adapter primers** | | |
| dt-AP | GGCCACGCGTCGACTAGTACTTTTTTTTTTTTTTTTT | Reverse transcription primer; |
| AP | GGCCACGCGTCGACTAGTAC | 3RACE nested-PCR; |
| dg-AP | GGCCACGCGTCGACTAGTACGGGGGGGGGG | 5RACE nested-PCR; |
