# Supplementary material for: Identification two novel nacrein-like proteins involved in the shell formation of the Pacific oyster Crassostrea gigas
Source: Mol Biol Rep. 2014 Mar 2;41(7):4273–8. doi: 10.1007/s11033-014-3298-z (PMC4066178; doi:10.1007/s11033-014-3298-z)
Supplement: Supplementary file 2 — Alignments of nine nacrein-like proteins from seven mollusc species and CA1 from Homo sapiens were edited using Bioedit software. Conserved amino acids have black (100%) or dark gray (70%) backgrounds, whereas nonconserved amino acids have a white background. The putative active sites of the functional domains in the CA1 (Homo sapiens) are indicated by diamonds (◊), among which 25 active sites appear in the nacrein-like proteins of C.gigas, including three histidine residues that bind to zinc ions(◊). Signal peptides and the transmembrane region in nacrein-like proteins F1 and F2 are boxed and highlighted in yellow, respectively. Amino acids in pink background indicate putative phosphorylation sites (F1: Ser 14, Thr 2, Tyr 4; F2: Ser 14, Thr 2, Tyr 12); Characters highlighted in green indicate putative N-glycosylation sites (F1: 103, 153, 156, 416; F2: 119, 124, 423). Accession numbers: Homo sapiens: CA1/ AAH27890; Crassostrea gigas: nacrein-like protein F1, F2 and F3; Pinctada maxima: nacrein like protein (pm) /BAF42330.1; Mizuhopecten yessoensis: nacrein-like protein P1/BAF42331.1; Pinctada fucata: nacrein/BAA11940.1; Pinctada margaritifera: nacrein-like protein C5/AEC03973; Mytilus californianus: nacrein-like protein/P86856.1 and Turbo marmoratus: nacrein/ BAB91157 (PDF 132 kb) [file 11033_2014_3298_MOESM2_ESM.pdf]

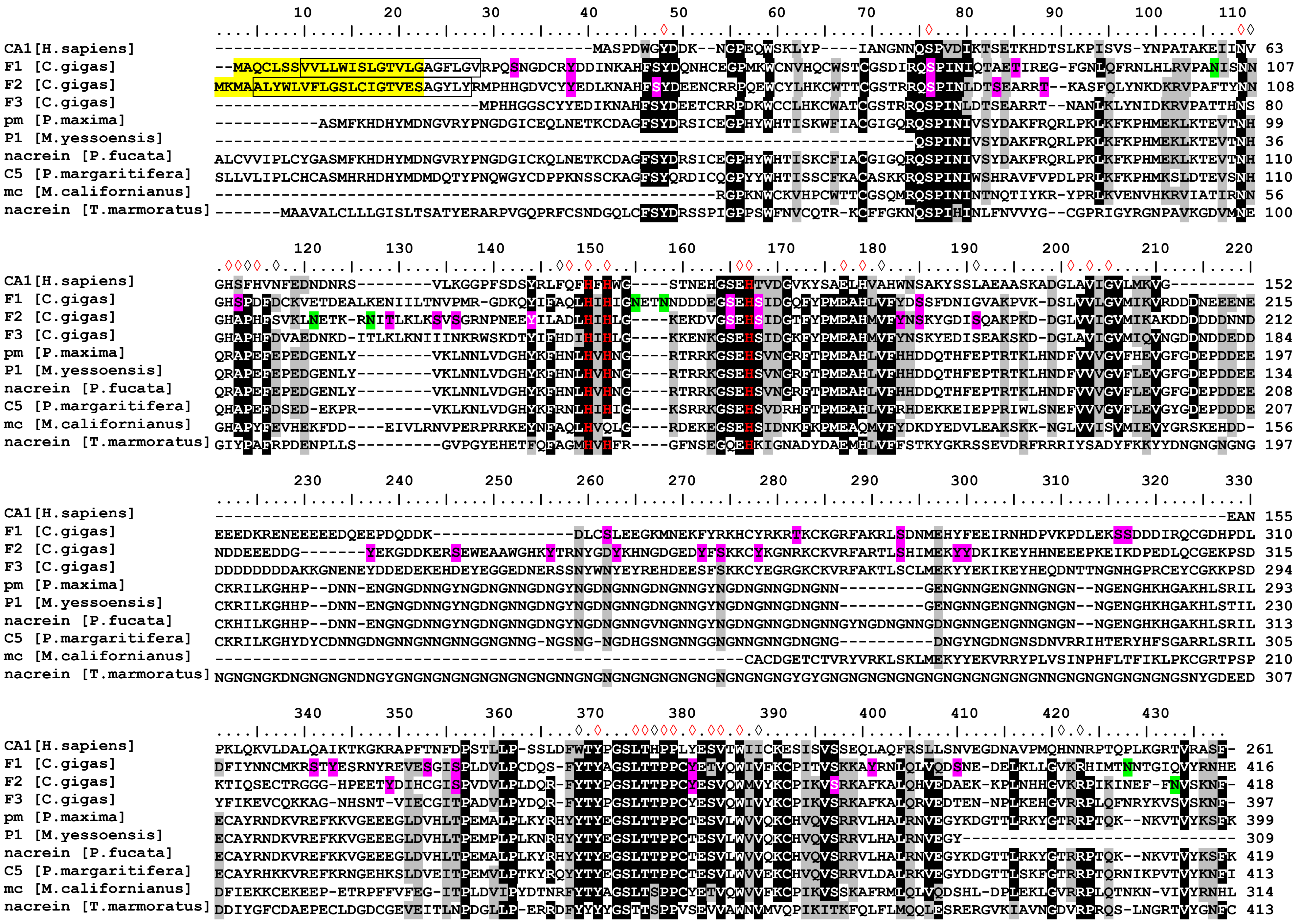

CA1[H.sapiens] --MASPDWGYDDK--NGPEQWSKLYP----IANGNNQSPVDIKTSETKHDTSLKPISVS-YNPATAKEIINV 63
F1 [C.gigas] --MAQCLSSVVLLWISLGTVLGAGFLGVRPQSNGDCRYDDINKAHFSYDQNHCEGPMKWCNVHQCWSTCGSDIRQSPINIQTAETIREG-FGNLQFRNLHLRVPANISNN 107
F2 [C.gigas] MKMAALYWLVFLGSLCIGTVESAGYLYRMPHHGDVCYYEDLKNAHFSYDEENCRRPQEWCYLHKCWTTCGSTRRQSPINLDTSEARRT--KASFQLYNKDKRVPAFTYNN 108
F3 [C.gigas] ----------------------------MPHHGGSCYYEDIKNAHFSYDEETCRRPDKWCCLHKCWATCGSTRRQSPINLDTSEARRT--NANLKLYNIDKRVPATTHNS 80
pm [P.maxima] -----------ASMFKHDHYMDNGVRYPNGDGICEQLNETKCDAGFSYDRSICEGPHYWHTISKWFIACGIGQRQSPINIVSYDAKFRQRLPKLKFKPHMEKLKTEVTNH 99
P1 [M.yessoensis] --QSPINIVSYDAKFRQRLPKLKFKPHMEKLKTEVTNH 36
nacrein [P.fucata] ALCVVIPLCYGASMFKHDHYMDNGVRYPNGDGICKQLNETKCDAGFSYDRSICEGPHYWHTISKCFIACGIGQRQSPINIVSYDAKFRQRLPKLKFKPHMEKLKTEVTNH 110
C5 [P.margaritifera] SLLVLIPLCHCASMHRHDHYMDMDQTYPNQWGYCDPPKNSSCKAGFSYQRDICQGPYYWHTISSCFKACASKKRQSPINIWSHRAVFVPDLPRLKFKPHMKSLDTEVSNH 110
mc [M.californianus] ---RGPKNWCKVHPCWTTCGSQMRQSPININTNQTIYKR-YPRLKVENVHKRVIATIRNN 56
nacrein [T.marmoratus] -------MAAVALCLLLGISLTSATYERARPVGQPRFCSNDGQLCFSYDRSSPIGPPSWFNVCQTR-KCFFGKNQSPIHINLFNVVYG--CGPRIGYRGNPAVKGDVMNE 100
CA1[H.sapiens] GHSFHVNFEDNDNRS--------VLKGGPFSDSYRLFQFHFHWG----STNEHGSEHTVDGVKYSAELHVAHWNSAKYSSLAEAASKADGLAVIGVLMKVG--------- 152
F1 [C.gigas] GHSPDFDCKVETDEALKENIILTNVPMR-GDKQYIFAQLHIHIGNETNNDDDEGSEHSIDGQFYPMEAHLVFYDSSFDNIGVAKPVK-DSLVVLGVMIKVRDDDNEEENE 215
F2 [C.gigas] GHAPHFSVKLNETK-RNITLKLKSVSGRNPNEEYILADLHIHLG----KEKDVGSEHSIDGTFYPMEAHMVFYNSKYGDISQAKPKD-DGLVVIGVMIKAKDDDDDDNND 212
F3 [C.gigas] GHAPHFDVAEDNKD-ITLKLKNIIINKRWSKDTYIFHDIHIHLG----KKENKGSEHSIDGKFYPMEAHMVFYNSKYEDISEAKSKD-DGLAVIGVMIQVNGDDNDDEDD 184
pm [P.maxima] QRAPEFEPEDGENLY--------VKLNNLVDGHYKFHNLHVHNG----RTRRKGSEHSVNGRFTPMEAHLVFHHDDQTHFEPTRTKLHNDFVVVGVFHEVGFGDEPDDEE 197
P1 [M.yessoensis] QRAPEFEPEDGENLY--------VKLNNLVDGHYKFHNLHVHNG----RTRRKGSEHSVNGRFTPMEAHLVFHHDDQTHFEPTRTKLHNDFVVVGVFLEVGFGDEPDDEE 134
nacrein [P.fucata] QRAPEFEPEDGENLY--------VKLNNLVDGHYKFHNLHVHNG----RTRRKGSEHSVNGRFTPMEAHLVFHHDDQTHFEPTRTKLHNDFVVVGVFLEVGFGDEPDDEE 208
C5 [P.margaritifera] QHAPEFDSED-EKPR--------VKLKNLVDGHYKFRNLHIHIG----KSRRKGSEHSVDRHFTPMEAHLVFRHDEKKEIEPPRIWLSNEFVVVGVFLEVGYGDEPDDDE 207
mc [M.californianus] GHAPYFEVHEKFDD----EIVLRNVPERPRRKEYNFAQLHVQLG----RDEKEGSEHSIDNKFKPMEAQMVFYDKDYEDVLEAKSKK-NGLVVISVMIEVYGRSKEHDD- 156
nacrein [T.marmoratus] GIYPAFRPDENPLLS---------GVPGYEHETFQFAGMHVHFR----GFNSECQEHKIGNADYDAEMHLVFFSTKYGKRSSEVDRFRRRIYSADYFKKYYDNGNGNGNG 197
CA1[H.sapiens] ---EAN 155
F1 [C.gigas] EEEDKRENEEEEEDQEEPDQDDK---------------DLCSLEEGKMNEKFYRKHCYRKRTKCKGRFAKRLSDNMEHYFEEIRNHDPVKPDLEKSSDDDIRQCGDHPDL 310
F2 [C.gigas] NDDEEEDDG-------YEKGDDKERSEWEAAWGHKYTRNYGDYKHNGDGEDYFSKKCYKGNRKCKVRFARTLSHIMEKYYDKIKEYHHNEEEPKEIKDPEDLQCGEKPSD 315
F3 [C.gigas] DDDDDDDDAKKGNENEYDDEDEKEHDEYEGGEDNERSSNYWNYEYREHDEESFSKKCYEGRGKCKVRFAKTLSCLMEKYYEKIKEYHEQDNTTNGNHGPRCEYCGKKPSD 294
pm [P.maxima] CKRILKGHHP--DNN-ENGNGDNNGYNGDNGNNGDNGYNGDNGNNGDNGNNGYNGDNGNNGDNGNN---------GENGNNGENGNNGNGN--NGENGHKHGAKHLSRIL 293
P1 [M.yessoensis] CKRILKGHHP--DNN-ENGNGDNNGYNGDNGNNGDNGYNGDNGNNGDNGNNGYNGDNGNNGDNGNN---------GENGNNGENGNNGNGN--NGENGHKHGAKHLSTIL 230
nacrein [P.fucata] CKHILKGHHP--DNN-ENGNGDNNGYNGDNGNNGDNGYNGDNGNNGVNGNNGYNGDNGNNGDNGNNGYNGDNGNNGDNGNNGENGNNGNGN--NGENGHKHGAKHLSRIL 313
C5 [P.margaritifera] CKRILKGHYDYCDNNGDNGNNGNNGNNGGNGNNG-NGSNG-NGDHGSNGNNGGNGNNGNNGDNGNG----------DNGYNGDNGNSDNVRRIHTERYHFSGARRLSRIL 305
mc [M.californianus] --CACDGETCTVRYVRKLSKLMEKYYEKVRRYPLVSINPHFLTFIKLPKCGRTPSP 210
nacrein [T.marmoratus] NGNGNGKDNGNGNGNDNGYGNGNGNGNGNGNGNGNNGNGNGNGNGNGNGNGNGNGNGNGYGYGNGNGNGNGNGNGNGNGNGNGNNGNGNGNGNGNGNGSNYGDEED 307
CA1[H.sapiens] PKLQKVLDALQAIKTKGKRAPFTNFDPSTLLP-SSLDFWTYPGSLTHPPLYESVTWIICKESISVSSEQLAQFRSLLSNVEGDNAVPMQHNNRPTQPLKGRTVRASF- 261
F1 [C.gigas] DFIYNNCMKRSTYESRNYREVESGISPLDVLPCDQS-FYTYAGSLTTPPCYETVQWIVFKCPITVSKKAYRNLQLVQDSNE-DELKLLGVKRHIMTNNTGIQVYRNHE 416
F2 [C.gigas] KTIQSECTRGGG-HPEETYDIHCGISPVDVLPLDQR-FYTYPGSLTTPPCYESVQWMVYKCPIKVSRKAFKALQHVEDAEK-KPLNHHGVKRPIKINEF-FNVSKNF- 418
F3 [C.gigas] YFIKEVCQKKAG-NHSNT-VIECGITPADVLPYDQR-FYTYPGSLTTPPCYESVQWIVYKCPIKVSRKAFKALQRVEDTEN-NPLKEHGVRRPLQFNRYKVSVSKNF- 397
pm [P.maxima] ECAYRNDKVREFKKVGEEEGLDVHLTPEMALPLKYRHYYTYEGSLTTPPCTESVLWVVQKCHVQVSRRVLHALRNVEGYKDGTTLRKYGTRRPTQK--NKVTVYKSFK 399
P1 [M.yessoensis] ECAYRNDKVREFKKVGEEEGLDVHLTPEMPLPLKNRHYYTYEGSLTTPPCTESVLWVVQKCHVQVSRRVLHALRNVEGY----------------------------- 309
nacrein [P.fucata] ECAYRNDKVREFKKVGEEEGLDVHLTPEMALPLKYRHYYTYEGSLTTPPCTESVLWVVQKCHVQVSRRVLHALRNVEGYKDGTTLRKYGTRRPTQK--NKVTVYKSFK 419
C5 [P.margaritifera] ECAYRHKKVREFKRNGEHKSLDVEITPEMVLPTKYRQYYTYEGSLTTPPCTESVLWVVEKCHVQVSRRVLDALRKVEGYDDGTTLSKFGTRRPTQRNIKPVTVYKNFI 413
mc [M.californianus] DFIEKKCEKEEP-ETRPFFVFEG-ITPLDVIPYDTNRFYTYAGSLTSPPCYETVQWVVFKCPIKVSSKAFRMLQLVQDSHL-DPLEKLGVRRPLQTNKN-VIVYRNHL 314
nacrein [T.marmoratus] DDIYGFCDAEPECLDGDCGEVEITLNPDGLLP-ERRDFYYYYGSTTSPPVSEVVAWNVMVQPIKITKFQLFLMQQLESRERGVKIAVNGDVRPRQS-LNGRTVYGNFC 413
